# Mast cells: “central regulatory hub” of neuro-endocrine-immune dysregulation in vitiligo

**DOI:** 10.3389/fimmu.2026.1808882

**Published:** 2026-06-26

**Authors:** Chen Zhang, Zhanhong Cao, Yuxin Bai, Xuanxuan Zhu, Yue Shen, Zhenhua Wang, Jincai Li, Wenjian Wang, Meng Zhang

**Affiliations:** 1 Bozhou University, Traditional Chinese Medicine College, Bozhou, Anhui, China; 2 Anhui Shimao Traditional Chinese Medicine Co., Ltd., Bozhou, Anhui, China

**Keywords:** endocrine system, immune system, mast cells, nervous system, vitiligo

## Abstract

Mast cells (MCs), as innate immune cells residing in cutaneous tissues, serve as critical mediators in the cross-talk between the neuroendocrine and immune systems. Upon activation by internal or external stimuli, MCs synthesize, accumulate, and secrete a variety of regulatory mediators, thereby exerting multifaceted effects through their secretory capacity and regulatory functions. Vitiligo, a complex disorder with a heterogeneous etiology encompassing genetic susceptibility, oxidative stress, autoimmunity, and neurogenic components, remains incompletely understood. Current pathogenetic models based solely on neural, humoral, or immunological frameworks fail to provide a comprehensive explanation for its development. Despite the incomplete characterization of mechanisms driving MC accumulation in vitiligo lesions, therapeutic agents targeting MCs and their signaling pathways (e.g., ketotifen, imatinib) have already been clinically implemented. Notably, MCs exhibit bidirectional interactions with neural and endocrine systems: they respond to neurotransmitter and hormonal signals while simultaneously releasing bioactive substances that reciprocally modulate neural activity, endocrine balance, and local immune responses, ultimately impacting melanocyte function. Consequently, targeting MCs and their key signaling pathways(e.g., the MrgX2-PAR2 axis and chymase-TLR4 pathway) represents a promising novel approach for vitiligo management, with potential applications in controlling disease progression, preventing relapse, and advancing therapeutic development.

## 1 Introduction

The skin is a sophisticated neuroendocrine organ, subject to regulation by the hypothalamic-pituitary-adrenal (HPA) axis of the central nervous system (1, 2). Under exposure to diverse stressors, cutaneous tissues engage neural, immune, and endocrine pathways to orchestrate physiological adaptations, including melanogenesis modulation. During systemic stress responses, dermal mast cells (MCs) undergo degranulation, releasing inflammatory mediators that establish these cells as a “central regulatory hub” (3). As key orchestrators of innate and adaptive immunity, MCs participate in intricate intercellular dialogues with neighboring cell types, thereby supporting epidermal barrier integrity and immune equilibrium. MCs act as local tissue sentinels. Coupled with their secretory versatility and regulatory influence, this makes them essential nodes for integrating and disseminating signals across the neuro-endocrine-immune network.

Vitiligo is a complex acquired skin disorder characterized by depigmentation resulting from the combined effects of multiple pathogenic factors involving various systems. Its etiology is intricate, with increasing incidence rates, severe consequences, and a trend toward younger onset (4). The pathogenesis of vitiligo involves genetic predisposition, oxidative stress, autoimmunity, and neurogenic factors. However, hypotheses proposed from neural, humoral, and immunological perspectives alone cannot fully explain the disease mechanism. One thing is certain: all proposed mechanisms ultimately culminate in melanocyte death or dysfunction (5, 6). Current vitiligo research rarely considers the involvement of MCs, and virtually no research has investigated their role in melanocyte dysfunction and death, which is a critical endpoint of the disease. This review therefore examines the role of MCs in the vitiligo microenvironment in disease progression.

## 2 The origin, classification, and activation of MCs

**Figure 1:**
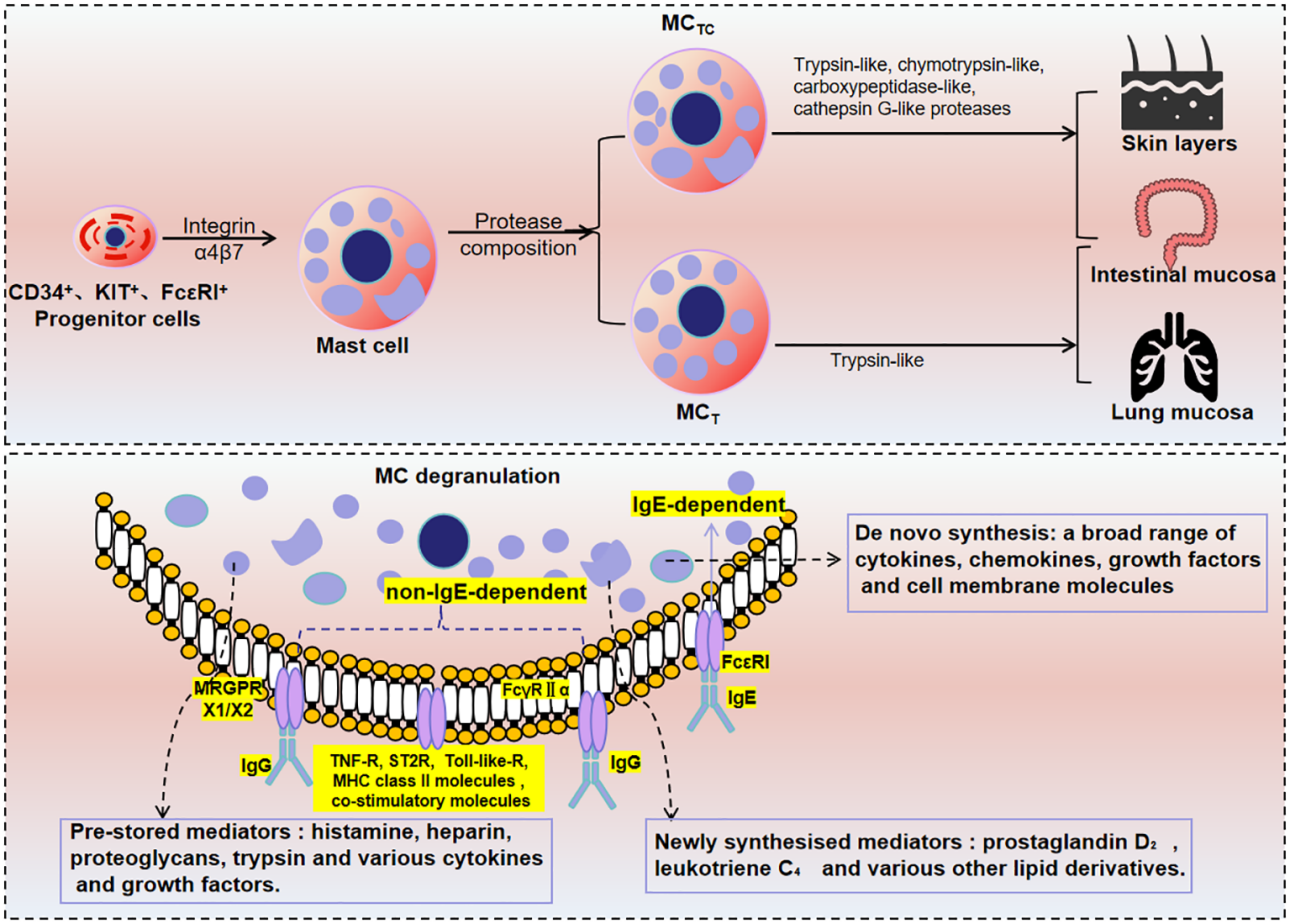
The origin, classification, and activation of MCs. (Mast cells originate from precursor cells that express CD34+, KIT+, and FcϵRI+. Based on protease composition, mast cells can be classified into two types: MCTC and MCT. There are two mechanisms of mast cell activation: one involves the binding of allergen-crosslinked IgE) to the high-affinity membrane receptor FcϵRI, and the other involves a non-IgE-dependent, receptor-mediated degranulation mechanism. The mast cell degranulation process generates and releases three main types of mediators: pre-stored mediators, newly synthesized mediators, and *de novo* synthesized mediators).

MCs originate from multipotent hematopoietic progenitors in the bone marrow and are derived from monocytes. Progenitor cells with mast cell differentiation potential are characterized by expression of CD34^+^, KIT^+^, and FcϵRI^+^ (7). Under the influence of microenvironmental factors, these precursors undergo terminal differentiation into mature cells, with integrin α4β7 facilitating their migration to peripheral tissues. Mature MCs function as tissue-resident immune cells capable of long-term strategic survival in localized sites such as skin, respiratory tract, and intestinal mucosa, with the skin exhibiting the highest enrichment of these cells (8). Human MCs are classified into two subtypes based on protease composition: MC_TC_ and MC_T_. MC_TC_ cells contain trypsin-like, chymotrypsin-like, carboxypeptidase-like, and cathepsin G-like proteases, predominantly localizing to subcutaneous skin layers and intestinal mucosa. In contrast, MC_T_ cells exclusively express trypsin-like proteases and are widely distributed in lung and intestinal mucosal tissues (9). There are two mechanisms of MCs activation. One mechanism is involves allergen-crosslinked IgE binding to the high-affinity membrane receptor FcϵRI, which triggers degranulation. The other mechanism is non-IgE-dependent, receptor-initiated degranulation via receptors such as FcγRIIα or Mas-related G protein-coupled receptors X1/X2 (MRGPRX1/X2) (10, 11). MCs degranulation produces and releases three main types of mediator: pre-stored mediators such as histamine, heparin, proteoglycans, trypsin and various cytokines and growth factors. Newly synthesized mediators include prostaglandin D_2_, leukotriene C_4_ and various other lipid derivatives. *De novo* synthesis encompasses a broad range of cytokines, chemokines, growth factors and cell membrane molecules, such as TNF receptors, IL-33 receptor ST2, Toll-like receptors, MHC class II molecules and co-stimulatory molecules. All of these factors and receptors play a crucial role in regulating inflammatory and immune responses in chronic diseases (12, 13)(**Figure 1**).

## 3 MCs expression in vitiligo pathogenesis

Recent studies have elucidated the pivotal role of MCs in vitiligo progression. Aroni K et al. reported significant differences in MCs density, CD34^+^ cell populations, and vascular endothelial growth factor (VEGF) expression between the central and peripheral regions of vitiligo-affected skin (P ≤ 0.001), with activated MCs in these areas exhibiting cytokine production capacity (14). In a murine model, acute auditory stimulation in C57BL/6J mice induced dermal MCs accumulation, accompanied by elevated substance P (SP)-positive nerve fiber density in proximity to activated MCs. This neuro-immune interaction was associated with follicular melanocyte apoptosis, suggesting a potential link between sensory stimuli and melanocyte loss (15). Further mechanistic insights revealed that acute sound stimulation under fear-conditioned stress activates dermal MCs, establishing a functional neuro-mast cell circuit that mediates stress memory formation in response to auditory cues (16). Chronic stress models, including chronic restraint stress (CRS) and chronic unpredictable mild stress (CUMS), demonstrated profound effects on melanogenesis. CRS preferentially activates cutaneous sensory neurons, leading to the massive release of substance P (SP) and the subsequent generation of SP17—a bioactive fragment that selectively triggers MRGPRX2-dependent mast cell degranulation, resulting in melanocyte damage (17). CUMS combined with monobenzone (MBEH) induces vitiligo through combined effects on neuronal signaling, mast cells, and melanocytes. CUMS disrupts skin neural homeostasis and promotes neuropeptide release, leading to the aggregation and degranulation of dermal mast cells; the resulting inflammatory microenvironment further inhibits melanin production and accelerates melanocyte death (18). Collectively, these findings highlight MCs as critical effectors in vitiligo pathogenesis, bridging neurogenic stress responses and melanocyte dysfunction.

## 4 Regulation of MCs and targeted cellular therapies in vitiligo

The significant upregulation and activation of MCs during the progression of vitiligo has attracted increasing attention from researchers. Studies indicate that resident cells such as keratinocytes, fibroblasts, and endothelial cells in the epidermis and dermis of affected areas of vitiligo patients significantly upregulate the expression of stem cell factor (SCF) (19). SCF serves as a potent chemotactic signal, guiding mast cell precursors from the peripheral blood to migrate and colonize lesion sites (20). Immunohistochemical analyses reveal markedly higher densities of SCF-positive cells in affected areas than in unaffected regions (21). Crucially, c-Kit is highly expressed on MCs surfaces. SCF binding activates multiple downstream pathways, including PI3K/Akt, MAPK and JAK/STAT. This enhances cellular sensitivity to injury signals and promotes the expression of anti-apoptotic proteins such as Bcl-2. Consequently, the MCs survival cycle is significantly prolonged (22). In Kit(W/Wv) mutant mouse models, c-Kit functional defects lead to a marked reduction in skin MCs numbers and diminished inflammatory responses (23). Interestingly, mast cells have been shown to synthesize and secrete SCF themselves, forming an autocrine or paracrine positive feedback loop whereby activated mast cells release SCF, which further attracts and activates additional mast cells and their progenitor cells, thereby amplifying local immune responses. This mechanism may explain the frequent observation of focal mast cell aggregates at the margins of vitiligo lesions, which are closely associated with disease progression. Beyond the SCF–c-Kit axis, multiple cytokines participate synergistically in mast cell chemotaxis and maintenance. For example, transforming growth factor-β (TGF-β), RANTES (CCL5) and stromal cell-derived factor-1 (SDF-1/CXCL12) have all been shown to effectively mediate the migration of MCs to specific tissue sites *in vitro* (24).

Although the mechanisms underlying MCs accumulation in vitiligo remain incompletely elucidated, targeting MCs and their associated signaling pathways has emerged as a promising therapeutic strategy for vitiligo and other chronic skin diseases. Currently, clinically used drugs that modulate MCs function primarily include antihistamines, MCs stabilizers, tyrosine kinase inhibitors, and corticosteroids. Among these, ketotifen, a classic MCs stabilizer, has been widely applied in both pediatric and adult vitiligo patients. Its primary mechanism involves inhibiting calcium ion influx, thereby preventing MCs degranulation and reducing the release of pro-inflammatory mediators, which alleviates local inflammation and slows the progression of pigment loss (25). Small-molecule tyrosine kinase inhibitors such as imatinib and ruxolitinib also demonstrate potential efficacy (26). Treatment with imatinib for myeloid leukemia can lead to hyperpigmentation, which is possibly related to the SCF-KIT gene (27). For melanocyte survival and migration, the spatiotemporally regulated expression of membrane-bound SCF and signal transduction through the c-kit receptor tyrosine kinase are key events at several stages of melanocyte development (28). Kunisada et al. reported that overexpression of soluble SCF in the mouse epidermis not only increased the number of melanocytes in the epidermis but also induced the accumulation of mast cells in the dermis (29). Han H et al. treated patients with GIST and vitiligo (acquired c-Kit mutations) using imatinib, resulting in GIST control and repigmentation of vitiligo (30). The specific mechanism involved inhibiting the mutated c-Kit while simultaneously activating normal melanocyte precursors. These findings suggest that imatinib, by targeting mast cells, has potential therapeutic utility for vitiligo. Ruxolitinib, on the other hand, inhibits the JAK1/2 pathway, attenuating downstream IFN-γ signaling and indirectly reducing the activation levels of MCs and other immune cells. Topical formulations targeting the JAK-STAT pathway have been approved for repigmentation therapy, further validating the central role of this pathway in immune regulation during disease progression (31, 32).

## 5 MCs function as the “central regulatory hub” in vitiligo

In the vitiligo microenvironment, the nervous, endocrine and immune systems are mobilized to regulate skin homeostasis. MCs remain present within the complex cellular and molecular networks of this microenvironment in the long term. As research into the local skin microenvironment deepens, the pivotal role of MCs as a key hub connecting the nervous, endocrine and immune systems in the progression of vitiligo has become increasingly evident.

### 5.1 MCs and the nervous system

MCs share functions with the nervous system, as they express typical neurotransmitters (histamine and serotonin) and a degranulation machinery that shares features with the neuronal apparatus at the synapse (33). Chronic psychological stress is a significant trigger or aggravating factor for vitiligo and can modulate the functional state of MCs via the sympathetic nervous system (SNS) (34).Under the stress, local skin nerve fibers release substances such as norepinephrine (NE), substance P (SP), etc., which act on key functional receptors expressed on the surface of mast cells, thereby further exacerbating the local inflammatory response. The key functional receptors expressed on mast cells responsible for sensing and receiving neuronal signals, mainly including neurokinin 1 receptor (NK1R), histamine H1 receptor, serotonin receptor and MAS-related G protein-coupled receptor-X2 (MRGPRX2 (35). Thus, MCs essentially act as “converters” that translate central emotional signals into peripheral skin immune dysregulation.

**Figure 2:**
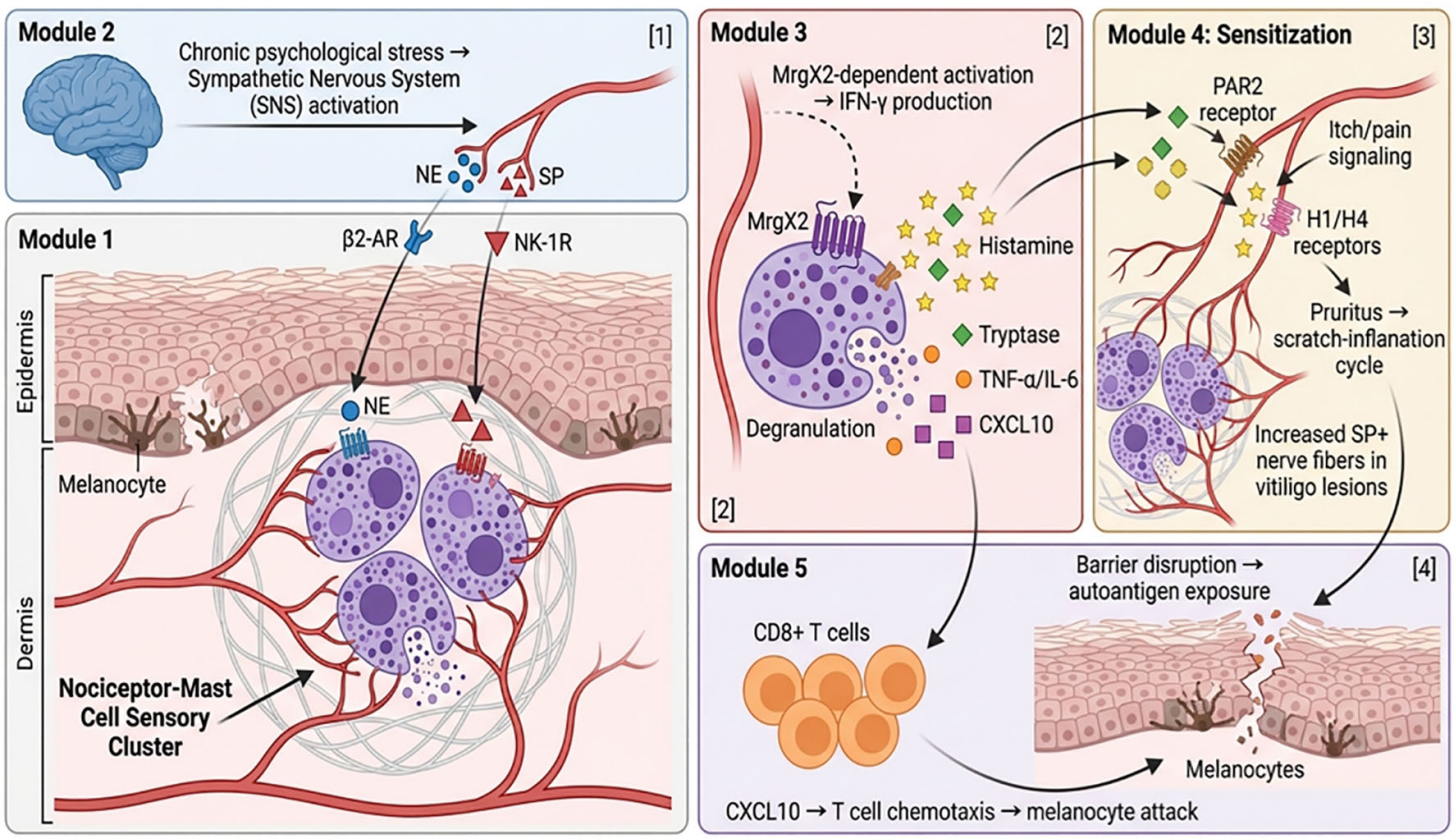
Bidirectional mast cells-neuron interactions drive neurogenic inflammation in vitiligo. (Chronic stress activates the sympathetic nervous system (Module 2), triggering release of norepinephrine (NE) and substance P (SP) from peripheral nerve terminals. These neurotransmitters prime dermal nociceptor-mast cell sensory clusters (Module 1) via β2-AR and NK-1R, respectively. Primed mast cells undergo MrgX2-dependent degranulation (Module 3), releasing histamine, tryptase, TNF-α, IL-6, and CXCL10. Mast cell mediators drive neuronal sensitization via PAR2 and H1/H4 receptors (Module 4), initiating an itch-scratch-inflammation cycle exacerbated by increased SP+ nerve fibers in lesions. CXCL10 recruits autoreactive CD8+ T cells to the skin (Module 5), which mediate melanocyte destruction).

In the pathological progression of vitiligo, MCs are not only key effector cells of immune responses but also central participants in a tightly coupled bidirectional interaction with the nervous system. Research indicates that MCs form highly specialized “nociceptor–mast cell sensory clusters” with sensory nerve endings in the skin (particularly unmyelinated nerve fibers), constituting a crucial anatomical basis for neuro-immune crosstalk (36). The functional units contain neuropeptides, such as substance P, vasoactive intestinal peptide (VIP) and calcitonin gene-related peptide (CGRP), which can activate the corresponding receptors (e.g. NK-1R, VPAC1/2) on the surfaces of MCs directly. This induces degranulation and the release of multiple pro-inflammatory mediators, including histamine, tryptase, TNF-α and IL-6 (37). These mediators in turn act on adjacent neurons. For example, tryptophan can trigger itching, burning pain, and neurosensitization by activating proteinase-activated receptor 2 (PAR2) on neurons; while histamine mediates intense itching through H1/H4 receptors, leading patients to repeatedly scratch, further damaging the skin barrier and promoting self-antigen exposure, forming a vicious cycle of “itch-scratch-inflammation” (38). This positive feedback mechanism is particularly active in the peripheral lesions of vitiligo, suggesting that mast cells-neurons play a key driving role in the lesion expansion phase. Furthermore, studies indicate that aging skin exhibits increased mast cell numbers with altered function, showing a greater tendency to aggregate around nerve fibers. This suggests age-related neural remodeling may influence mast cell distribution and activity (39). Accumulating evidence establishes MrgX2 as a master regulator of neuro-immune crosstalk in chronic inflammatory skin disorders, including vitiligo. In lesional skin, MrgX2-expressing mast cells form tight contacts with SP+ nerve fibers, enabling rapid responses to neuronal activation (40). Recent studies reveal abundant IFN-γ-producing MCs in the skin lesions of active vitiligo patients. Their activation depends on the MrgX2 receptor pathway, significantly enhancing Th1-type immune responses, promoting CXCL10 expression, and recruiting more CD8^+^ T cells to attack melanocytes (41). This dual role-amplifying neuronal excitability and driving adaptive immunity-renders MrgX2 a critical link between psychological stress, neural dysregulation, and autoimmune melanocyte destruction in vitiligo. MrgX2 serves as a key receptor linking nerves and MCs, suggesting this pathway may integrate dual functions of neural signal input and immune output.(**Figure 2**).

### 5.2 MCs and the endocrine system

Chronic psychological stress, a well-established precipitant and exacerbating factor in vitiligo, modulates MCs activity via central neuroendocrine pathways, primarily the HPA axis (42). Stress-induced activation of the HPA axis culminates in the systemic release of glucocorticoids (GCs), epinephrine (EPI), and norepinephrine (NE). These hormones profoundly alter MC functional states by shifting degranulation thresholds and skewing cytokine secretion profiles-typically promoting the release of pro-inflammatory factors (e.g., IL-6, IL-8) while suppressing anti-inflammatory mediators like IL-10, thereby tipping the local immune balance toward a chronic inflammatory state (43).

In the pathogenesis of vitiligo, MCs not only participate in inflammatory responses as immune effector cells but also form close interactions with the endocrine system within the local skin microenvironment through extensive receptor expression and secretory functions, becoming key nodes integrating the neuro-endocrine-immune network. Research indicates that catecholamines released by the sympathetic nervous system, such as norepinephrine and epinephrine, activate β2-adrenergic receptors (β2-AR) on mast cell surfaces. Through the cAMP-PKA signaling pathway, they regulate mast cell degranulation and cytokine secretion, initiating and sustaining Th1-type immune responses within the vitiligo microenvironment (43, 44). MCs also express glucocorticoid receptors (GR). Physiological concentrations of glucocorticoids typically exert inhibitory effects; however, under chronic stress or local hormone resistance states, this negative feedback mechanism may fail, leading to sustained MCs hyperreactivity (45–47). Furthermore, MCs actively participate in local hormone metabolism and functional regulation by secreting specific enzymes or binding proteins. For example, mast cell-derived aromatase may contribute to estrogen synthesis within the skin, and estrogen, via the ERβ receptor, protects melanocytes from oxidative stress damage (48). Simultaneously, mast cells release mediators like histamine and tryptophanase, indirectly influencing signaling pathways of pigment-related hormones such as melatonin and α-MSH. Among these, α-MSH exhibits anti-inflammatory and antioxidant properties while promoting melanogenesis. However, a mast cell-mediated chronic inflammatory environment may disrupt the binding efficiency of α-MSH to its receptor MC1R, thereby diminishing its protective effects (49).

### 5.3 MCs and the immune system

**Figure 3:**
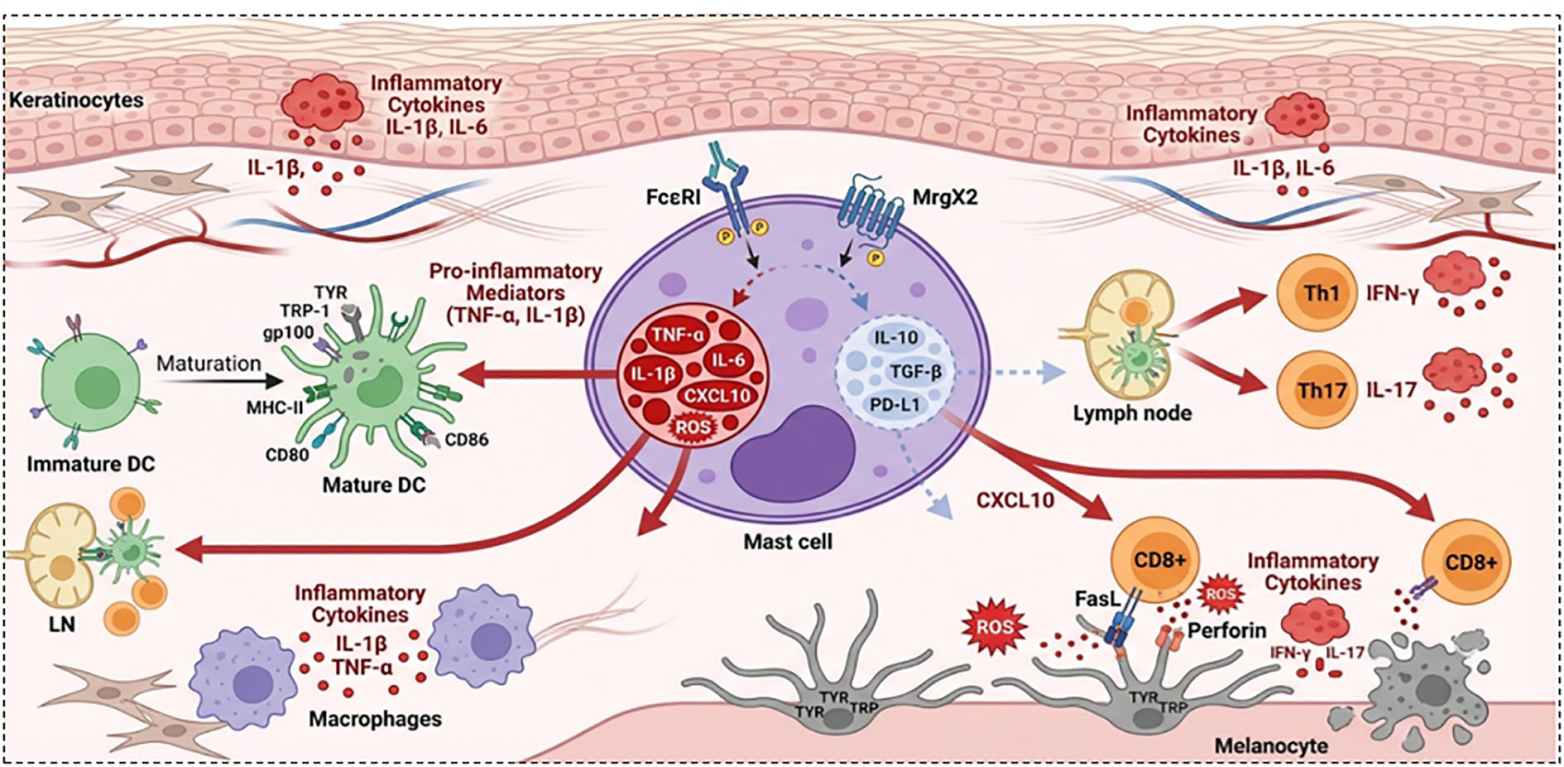
Mast cells orchestrate innate-adaptive immune crosstalk in vitiligo microenvironment. (Mast cells and the immune system interact in vitiligo in the following ways: 1. Mast cells degranulate to produce various cytokines, recruiting T cells to attack melanocytes; 2. They participate in the maturation and migration of dendritic cells (DCs), modulating the characteristics of antigen-presenting cells and regulating the local immune microenvironment; 3. They influence the pro-inflammatory and anti-inflammatory balance, thereby affecting the microenvironment of vitiligo).

MCs serve as a bridge between innate and adaptive immunity, modulating the properties of antigen-presenting cells and playing a crucial role in regulating the local immune microenvironment (**Figure 3**). In vitiligo, CD8^+^ cytotoxic T cells are key effector cells responsible for melanocyte destruction (50, 51). Research into autoimmune skin diseases has revealed that psoriasis patients have an increased number and frequency of mast cells and CD8 T cells in their dermal tissue, as well as elevated levels of mast cell degranulation (52). Mast cell degranulation releases multiple cytokines including IFN-γ, chemokine CXCL-10, as well as pro-inflammatory factors IL-6 and TNF-α, which recruit T cells to attack melanocytes (41, 53). Additionally, MCs participate in the maturation and migration of dendritic cells (DCs), subsequently affecting T cell differentiation pathways. TNF-α, IL-1β, and IL-6 secreted by activated MCs promote the phenotypic maturation of immature DCs, enhancing the expression of co-stimulatory molecules (e.g., CD80/CD86) and MHC class II molecules on their surface, thereby strengthening their antigen-presenting capacity. These mature DCs subsequently migrate to regional lymph nodes, presenting melanocyte-associated antigens (e.g., TYR, TRP-1, gp100) to naive T cells, driving their polarization toward Th1 and Th17 subsets (54, 55).

Additionally, MCs exhibit dual-edged sword properties in immunoregulation. Beyond pro-inflammatory functions, they secrete anti-inflammatory factors like IL-10 and TGF-β and express immune checkpoint molecules such as PD-L1, suppressing excessive immune responses or modulating regulatory T cell (Treg) function (56). However, in the context of vitiligo, this immunosuppressive capacity is often diminished, leading to a dominant pro-inflammatory phenotype. As tissue-resident immune cells, MCs exhibit significant immune cell infiltration in vitiligo lesions. They form complex interaction networks with other immune cells (e.g., macrophages, T cells), enabling them to sense changes in the tissue microenvironment and remodel the local immune microenvironment by releasing multiple mediators (57). For example, reactive oxygen species and inflammatory mediators released by MCs may exacerbate oxidative stress, thereby accelerating melanocyte death (58).

## 6 Summary

**Figure 4:**
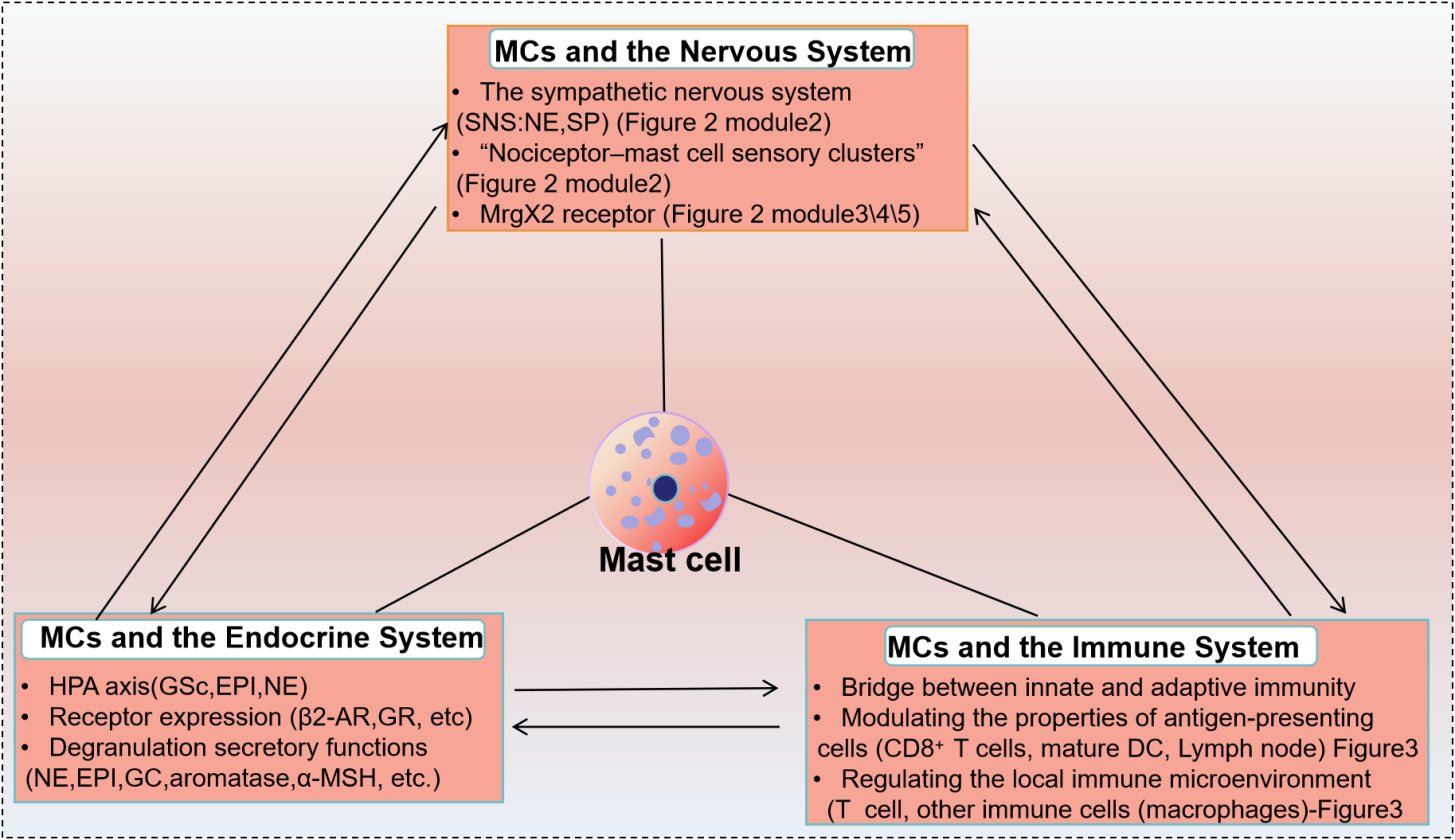
Mast cells: “central regulatory hub” of neuro-endocrine-immune dysregulation in vitiligo. (Mast cells act as a central regulatory hub, primarily in three key areas: 1. Mast cells and the nervous system. The sympathetic nervous system, “Nociceptor-mast cell sensory- clusters” MrgX2 receptor; 2. Mast cells and the endocrine system. HPA axis, Receptor expression, Degranulation and secretory functions; 3. Mast cells and the immune system. Bridge between innate and adaptive immunity, Modulating the properties of antigen-presenting cells, Regulating the local immune microenvironment).

Owing to their strategic anatomical positioning, diverse receptor repertoire, and potent secretory capability, MCs function as a pivotal nexus within the vitiligo lesion microenvironment, integrating signals across the neural, endocrine, and immune axes (**Figure 4**). They are not merely passive responders to neurotransmitters and hormones but active participants in a bidirectional dialogue, releasing a plethora of mediators that reciprocally modulate neuronal activity and endocrine tone while fundamentally reshaping the local immunological landscape. This orchestrated dysregulation culminates in melanocyte dysfunction and their progressive depletion. Consequently, MCs act as a “key amplifier” rather than a “core driver” in the pathogenesis of vitiligo, and targeting MCs and their key signaling pathways (e.g., the MrgX2-PAR2 axis and chymase-TLR4 pathway) represents a promising novel approach for vitiligo management, with potential applications in controlling disease progression, preventing relapse, and advancing therapeutic development. Future research must delineate the functional heterogeneity of distinct MCs subpopulations across various vitiligo stages and disease subtypes. Furthermore, elucidating the dynamic interaction networks between MCs and other skin-resident cells, including keratinocytes and fibroblasts, will be crucial for developing targeted, precision medicine approaches that effectively restore cutaneous immune homeostasis and melanocyte integrity.
